# Priming with FGF2 stimulates human dental pulp cells to promote axonal regeneration and locomotor function recovery after spinal cord injury

**DOI:** 10.1038/s41598-017-13373-5

**Published:** 2017-10-18

**Authors:** Kosuke Nagashima, Takahiro Miwa, Hitomi Soumiya, Daisuke Ushiro, Tomoko Takeda-Kawaguchi, Naritaka Tamaoki, Saho Ishiguro, Yumi Sato, Kei Miyamoto, Takatoshi Ohno, Masatake Osawa, Takahiro Kunisada, Toshiyuki Shibata, Ken-ichi Tezuka, Shoei Furukawa, Hidefumi Fukumitsu

**Affiliations:** 1 Laboratory of Molecular Biology, Department of Biofunctional Analysis, Gifu Pharmaceutical University, 1-25-4 Daigakunishi, Gifu, 501-1196 Japan; 2 Department of Oral and Maxillofacial Science, Gifu University Graduate School of Medicine, 1-1 Yanagido, Gifu, 501-1194 Japan; 3 Department of Orthopaedic Surgery, Gifu University Graduate School of Medicine, 1-1 Yanagido, Gifu, 501-1194 Japan; 4 Department of Regeneration Technology, Gifu University Graduate School of Medicine, 1-1 Yanagido, Gifu, 501-1194 Japan; 5 Department of Tissue and Organ Development, Gifu University Graduate School of Medicine, 1-1 Yanagido, Gifu, 501-1194 Japan; 6 Present Address: National Cancer Institute, National Institutes of Health (NIH), Bethesda, Maryland 20892 USA; 7 Present Address: Department of Orthopaedic Surgery and Spine Center, Gifu Municipal Hospital, 7-1 Kashima, Gifu, 500-8323 Japan; 8 Present Address: Department of Orthopaedic Surgery, Gifu Red Cross Hospital, 3-36 Iwakura, Gifu, 502-0844 Japan

## Abstract

Human dental pulp cells (DPCs), adherent cells derived from dental pulp tissues, are potential tools for cell transplantation therapy. However, little work has been done to optimize such transplantation. In this study, DPCs were treated with fibroblast growth factor-2 (FGF2) for 5–6 consecutive serial passages and were transplanted into the injury site immediately after complete transection of the rat spinal cord. FGF2 priming facilitated the DPCs to promote axonal regeneration and to improve locomotor function in the rat with spinal cord injury (SCI). Additional analyses revealed that FGF2 priming protected cultured DPCs from hydrogen-peroxide–induced cell death and increased the number of DPCs in the SCI rat spinal cord even 7 weeks after transplantation. The production of major neurotrophic factors was equivalent in FGF2-treated and untreated DPCs. These observations suggest that FGF2 priming might protect DPCs from the post-trauma microenvironment in which DPCs infiltrate and resident immune cells generate cytotoxic reactive oxygen species. Surviving DPCs could increase the availability of neurotrophic factors in the lesion site, thereby promoting axonal regeneration and locomotor function recovery.

## Introduction

Severe spinal cord injury (SCI) results in complete motor and sensory paralysis. The number of Japanese patients living with SCI is more than 100,000 and several million worldwide^1^. Spontaneous axonal regeneration does not occur in the adult mammalian central nervous system, including the spinal cord, and no effective systematic treatments are currently available for SCI patients. Accumulating evidence from basic research has elucidated molecular and cellular mechanisms of nerve regeneration in the SCI^2^. On the other hand, the observed effects of various treatments in clinical studies, including methylprednisolone^3^ and cell transplantation^4,5^, were quite limited and have not provided any definite conclusion. Thus, more effective methods/optimizations are being explored for use in SCI treatment.

Dental pulp cells (DPCs) are adherent cell types that arise from dental pulp tissues. These cell populations contain many types of neural-crest–derived ecto-mesenchymal stem cells and dental-pulp–derived stem cells (DPSCs), most of which express mesenchymal stem cell markers without endothelial/hematopoietic markers^6^. Using a rodent SCI model, DPCs/DPSCs transplantation was recently reported to induce more effective functional recovery than bone marrow-derived stromal cells or mesenchymal stem cell (BMSC) transplantation^6^ and are expected to be a promising cellular therapy for SCI^7,8^. Certain routes of administration and treatment in combination with growth factors and biomaterials have been reported to enhance the effects of BMSC transplantation on functional recovery in rat SCI models^9–11^. However, little work has been done to optimize human DPC transplantation to treat SCI.

One candidate growth factor for promoting the effects of DPC transplantation is fibroblast growth factor-2 (FGF2), as it is known to promote the survival and proliferation of multiple types of cells and to enhance angiogenesis; thus, FGF2 has attracted the attention of researchers in the field of regenerative medicine^12^. The following previous observations prompted us to investigate the effects of FGF2 on transplanted DPCs: (1) FGF2 promotes the proliferation of DPCs^13^; (2) FGF2 administration improves the recovery of locomotor function in rodent SCI models via proliferation of endogenous glial cells and fibronectin-positive cells^14,15^; (3) angiogenesis plays an important role in the function recovery of SCI, and FGF2 enhances DPSC transplantation-induced angiogenesis in subcutaneous tissues^16^.

To determine the effects of FGF2 on DPC transplantation, we injected DPCs pre-treated with FGF2 into the injury site immediately after complete transection of the rat spinal cord. DPC-transplanted rats with and without FGF2 pre-treatment of transplanted cells were compared with respect to DPC survival, axon regeneration, and recovery of motor function.

## Results

### Characterization of dental pulp cells treated with FGF2

**Figure 1:**
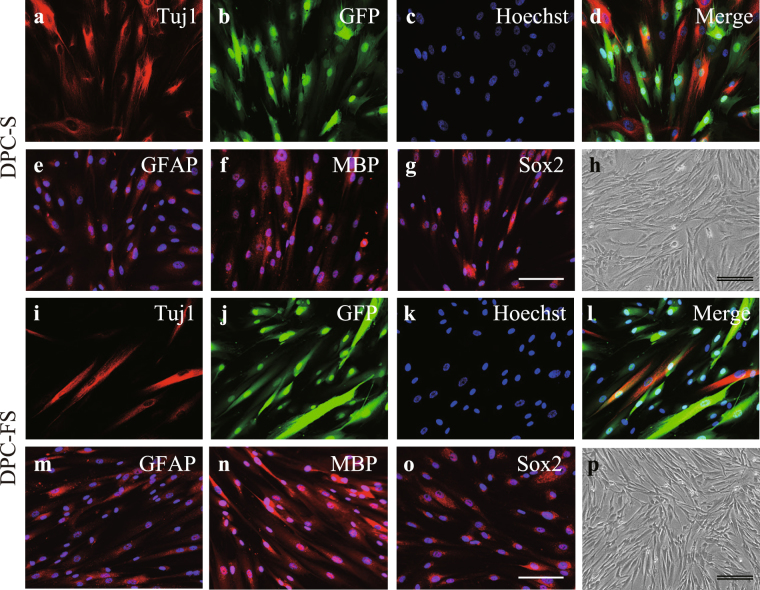
Morphology and expression of neural marker proteins of DPCs. DPCs were transfected with GFP reporter gene using a lenti-viral vector and cultured in the absence or presence of FGF2 (**a**–**h**. DPC-S or **i**–**p**. DPC-FS, respectively). Cultured DPCs were characterized immunohistochemically using antibodies against GFP (green; **b**,**d**,**j**,**l**) and/or each indicated neural cell marker (red), respectively: anti-Tuj1- (**a**,**d**,**i**,**l**), anti-GFAP- (**e**,**m**), and anti-MBP- (**f**,**n**) and anti-Sox2- (**g**,**o**) antibody, respectively. Nuclei were stained with Hoechst 33342 (blue; **c**–**g**,**k**–**o**). All DPCs showed spindle-shaped fibroblast morphologies (**h**,**p**). Scale bar, 100 μm; double bars, 200 μm.

After lentivirus-mediated green fluorescent protein (GFP) gene transfer and subculturing 6 times over 16–18 days in the presence and the absence of FGF2, the DPCs were examined for morphology and expression of neural markers and GFP (DPC-FS and DPC-S, respectively). All DPCs were similar in morphology when the cells were subconfluent (Fig. 1h and p): however, when close to confluence, the morphology of the DPC-FS changed to a long, spindle shape. Immunocytochemical analysis revealed that nearly all of the DPCs were labeled with GFP and expressed the neural lineage markers SRY-box containing gene 2 (Sox2, stem/progenitor cells), neuro-specific class III β-tubulin (Tuj1, premature and mature neuron), glial fibrillary acidic protein (GFAP, astrocyte), and myelin basic protein (MBP, oligodendrocyte) (Fig. 1 and Table 1). The expression of these markers and fraction of GFP-labeled cells were comparable between DPC-S and DPC-FS.

**Table 1:** Expression of neural marker proteins in cultured dental pulp cells (DPCs).

	DPC-S (%)	DPC-FS (%)	P
Sox2	98.8 ± 0.5	97.4 ± 0.5	0.15
Tuj1	42.9 ± 2.8	38.4 ± 1.4	0.17
GFAP	98.1 ± 0.2	97.7 ± 0.2	0.75
MBP	98.5 ± 0.2	96.8 ± 0.5	0.14
GFP	99.7 ± 0.2	99.9 ± 0.1	0.35

The percentages of cells positive for individual neural markers or GFP were evaluated with respect to the total number of cells. The values are expressed as the mean ± SE. Significant differences between the two groups were determined by Student’s *t*-test (n = 4).

### Priming with FGF2 promoted DPC-induced improvement in the locomotor function of SCI rats

**Figure 2:**
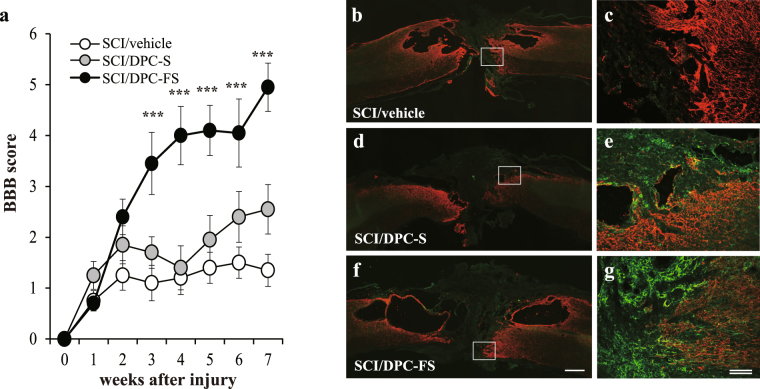
Effect of DPC-S or DPC-FS transplantation on the locomotor function of rats with complete-transected spinal cord and on axonal regeneration in the injury sites. (**a**) Locomotor function of the hind limbs was evaluated weekly for 7 weeks after injury. The values are expressed as the mean ± SE (n = 10 for each group). Significant differences from the control group (spinal cord injured rat with PBS injection) were determined using two-way repeated measure ANOVA with post hoc Tukey’s multiple comparison test. ***p < 0.0001; n = 10 for each group. (**b**–**g**) Fluorescence photomicrographs of sagittal spinal cord sections of SCI/DPC-S (**d**,**e**), SCI/DPC-FS (**f**,**g**) transplantation, or with PBS-injection (**b**,**c**) as a relative control. Sections immunostained for GAP-43 (green) and GFAP (red) were merged. Boxed areas in (**b**,**d**,**f**) are enlarged in (**c**,**e**,**g**), respectively. Note that the number of GAP-43–positive regenerative axons penetrating into GFAP-positive caudal tissues of rat spinal cord was higher with DPC-FS and lower with DPC-S transplantation. Such GAP-43–positive axons were rarely detected in the spinal cord of the relative control. Scale bar, 500 μm; double bars, 100 μm.

To examine the effects of DPC transplantation into SCI rats, locomotor functions were assessed weekly until 7 weeks post-lesion using the BBB locomotor rating scale, a commonly-used test of motor function after SCI. All the lesioned rats exhibited complete hindlimb paralysis immediately after injury (Fig. 2a). Two-way repeated measure analysis of variance (ANOVA) revealed a significant difference between groups (F [2, 27], 12.87; p = 0.0001) and between weeks after injury/transplantation (F [7, 189], 32.62; p < 0.0001), with interaction between groups and the weeks after injury/transplantation [F (14, 189), 6.325; p < 0.0001]. Post hoc analysis using Tukey’s multiple comparisons test indicated that transplantation of DPC-FS markedly ameliorated SCI-induced hindlimb paralysis (Fig. 2a). Vehicle-injected SCI rats without cell transplantation (SCI/vehicle group) and SCI rats transplanted with DPC-S (SCI/DPC-S group) exhibited no significant improvement in hindlimb motor function throughout the study (Fig. 2a). In contrast, rats undergoing DPC-FS transplantation (SCI/DPC-FS group) exhibited significantly improved locomotor recovery starting at 3 weeks after SCI compared to that of SCI/vehicle and SCI/DPC-S group (Fig. 2a).

### Priming with FGF2 stimulated DPC-induced axonal regeneration in the injured spinal cord

At 7 weeks after injury, large cavities and tissue loss were observed in the lesions of rats in the SCI/vehicle group. The cavities were surrounded by GFAP-positive astrocytes. Similar extents of cavitation and astrocytic glial scar formation were found in the lesions of the SCI/DPC-S and SCI/DPC-FS rats. Few axons with immunoreactivity toward growth-associated protein-43 (GAP-43), a specific marker for nerve regeneration, were detected in the SCI/vehicle group (Fig. 2b and c). However, a few GAP-43–positive axons were observed within lesions of SCI/DPC-S rats (Fig. 2d and e), and substantial numbers were observed in those of SCI/DPC-FS rats (Fig. 2f and g). In addition, no tumors, teratomas, or non-neuronal tissue formation were observed in any of the SCI groups (data not shown).

**Figure 3:**
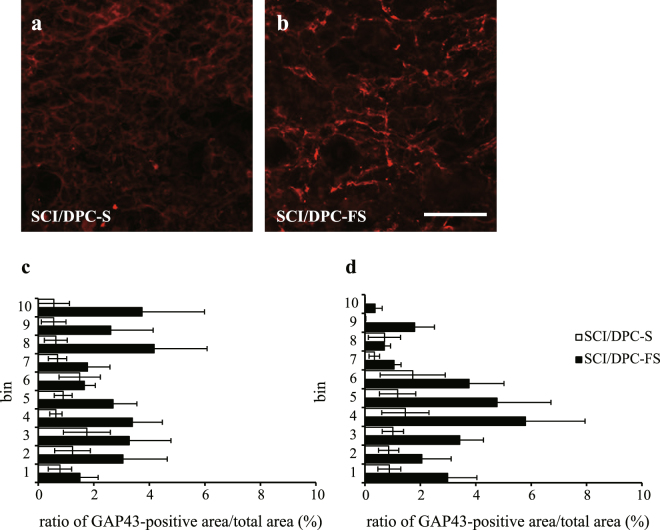
Distribution and quantification of GAP-43–positive area in the spinal cord at 1 week after SCI and DPC transplantation. Fluorescence photomicrographs were GAP-43–immunostained sagittal sections of the spinal cords at 1 mm caudal to the lesion site of the SCI rats at 1 week after transplantation of DPC-S (**a**) and DPC-FS (**b**), respectively. Percentages of GAP-43–positive area against total area were evaluated and the values were expressed as means ± SE (**c**, rostral and **d**, caudal). The ratio of GAP-43–positive area/total area were significantly larger in the SCI/DPC-FS than those in the SCI/DPC-S group (2.8 ± 0.5 vs 1.0 ± 0.4, for rostral, and 2.9 ± 0.7 vs 1.2 ± 0.5 for caudal; p < 0.05, and p < 0.05, respectively, Student’s *t*-test, n = 6 for SCI/DPC-S, and n = 9 for SCI/DPC-FS group, respectively). Scale bar 100 μm.

The initial phase of the axonal regeneration was examined at 7 days after spinal cord injury and DPC transplantation. As expected, GAP-43–positive axons were barely detected in the SCI/vehicle groups (data not shown). However, small but significant numbers of GAP-43–positive axon-like processes were randomly extended in both the caudal and rostral areas around the lesions of the SCI/DPCs rats. Representative photographs from the caudal area of the longitudinal sections of SCI/DPC-S and SCI/DPC-FS are shown in Fig. 3a and b, respectively. Quantitative analysis revealed that the number of GAP-43–positive axons observed in lesions was approximately 3.0-fold higher in SCI/DPC-FS rats than in SCI/DPC-S rats (Fig. 3c and d).

Since FGF2-primed DPCs promoted axonal regeneration, it is possible that FGF2 increases DPC production of neurotrophic factors that facilitate axonal regeneration. To address this possibility, cultured cortical neurons were labeled by lentivirus-mediated GFP gene transfer at 2 days *in vitro* (DIV). Following treatment of DPC-conditioned medium from 2 to 4 DIV, the cultures were processed for immunostaining with anti–microtubule-associated protein 2 (MAP2; marker of mature neurons) antibodies; the morphologies of MAP2/GFP double-positive neurons were evaluated. Sholl analysis^17,18^ with one-way ANOVA revealed a significant difference between groups (F [2, 54] = 5.336; p < 0.01). Post hoc analysis with Tukey’s multiple comparisons test indicated that medium conditioned by high-density culture of DPCs significantly increased the number of neurites crossing at radial distances of 100 μm (Supplementary Fig. S1c) but not 50 μm (Supplementary Fig. S1b). The neurite-extension–promoting activity was comparable between the media conditioned by DPC-S and DPC-FS.

To address which kinds of neurotrophic factors were produced by DPCs, we first examined the mRNA expression of several neurotrophic factors in DPC-S and DPC-FS cells. Real-time quantitative PCR revealed that FGF2 treatment caused an approximately 3.3-fold decrease and 3.8-fold increase in the expression of NT3 and VEGF mRNA, respectively, whereas the expression of BDNF and GDNF mRNA was not altered by FGF2 treatment (Table 2). Next, we assessed the production of neurotrophic factors/growth factors at the protein level. Cell lysates were collected for membrane-based human growth factor antibody array detection. A pilot study for this assay indicated that DPCs could produce high levels of FGF2 and HGF compared with the other neurotrophic factors/growth factors (Supplementary Fig. S2). Based on these results, the production of several neurotrophic factors by DPCs including BDNF, GDNF, FGF2, and HGF was examined by western blot analysis. The production of BDNF was slightly but not significantly higher (Supplementary Fig. S3a and b) and HGF was twofold higher (Supplementary Fig. S3a and e) in DPC-FS than in DPC-S, whereas the production of GDNF (Supplementary Fig. S3a and c) and FGF2 (Supplementary Fig. S3a and d) was comparable in both cell types. Production and secretion of mature BDNF were also confirmed in DPCs by enzyme-linked immunosorbent assay (ELISA; Cell lysates: DPC-S, 67.6 ± 7.2 and DPC-FS, 124.7 ± 9.6 pg/mL, *p* < 0.005; Conditioned medium: DPC-S, 45.2 ± 2.6 and DPC-FS, 51.2 ± 8.4 pg/mL, *p* = 0.52; Student’s *t*-test, *n* = 5).

**Table 2:** Messenger RNA expression of neurotrophic factors.

	DPC-S	DPC-FS	P
BDNF	1.0 ± 0.1	1.2 ± 0.2	0.23
NT-3	1.0 ± 0.0	0.3 ± 0.0	<0.001
GDNF	1.0 ± 0.0	0.9 ± 0.1	0.08
VEGF	1.0 ± 0.0	3.8 ± 0.1	<0.001

The ratio of mRNA expression in DPC-S for each neurotrophic factor was averaged, and the mean value was standardized to 1.0 for comparison with DPC-FS. The values are expressed as the mean ± SE. Significant differences between the two groups were determined by Student’s *t*-test (n = 6).

### Priming with FGF2 improves DPC viability in the injured spinal cord after transplantation

**Figure 4:**
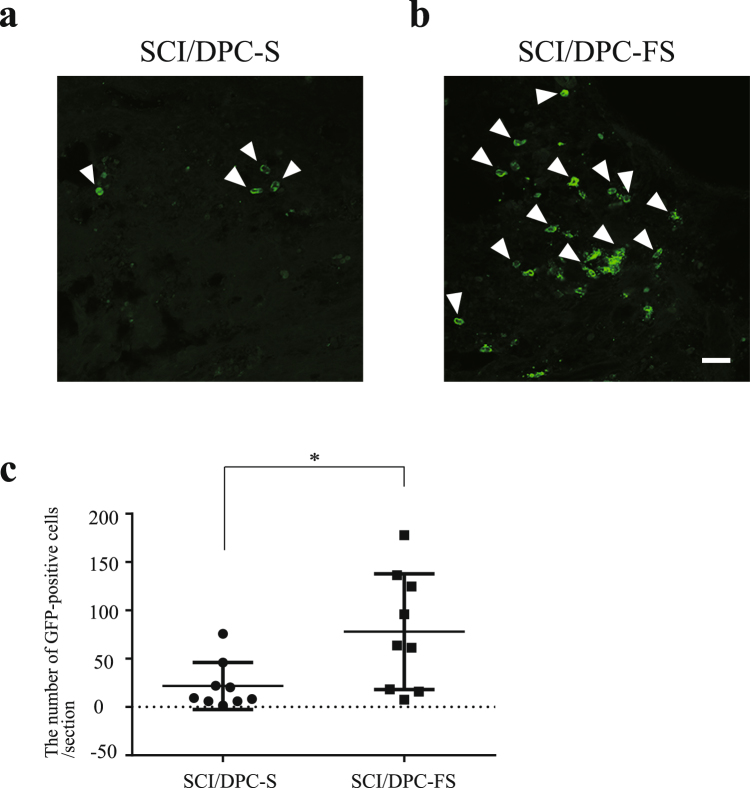
Distribution and quantification of the DPCs in the spinal cord at 7 weeks after SCI and transplantation. Fluorescence photomicrographs were GFP-immunostained sagittal sections of the spinal cords of the SCI/DPC-S (**a**) and SCI/DPC-FS group (**b**), at 7 weeks after transplantation, respectively: The GFP positive cells (green) were indicated by arrows. Scale bar 50 μm. (**c**) The number of the GFP-positive cells in the spinal cord at 7 weeks after DPCs transplantation: The values were expressed as means ± SE indicated by horizontal bars. Significant difference between the two groups was determined by Student’s *t*-test. *p < 0.05, n = 8. Note that the FGF2 priming increased in the number of GFP-positive cells in the spinal cord of SCI at 7 weeks after transplantation.

To determine whether the FGF2 priming influences DPC survival after cell transplantation, the distribution and number of GFP-positive cells were examined in the spinal cords of SCI/DPC-S and SCI/DPC-FS rats at 7 weeks after injury/transplantation. Immunohistochemical analysis revealed as low number of GFP-positive cells in the spinal cord at 7 weeks after transplantation, regardless of FGF2 priming. Quantitative analysis revealed that the number was less than 1% of the total transplanted cells; 30–90 cells per 25-μm thick section were equal to 0.36–1.08% of transplanted cells. In contrast, the number of the GFP-positive cells increased significantly, approximately 3.0–4.0- fold, with FGF2 priming (Fig. 4a and b). Most of the DPCs were localized close to the lesion site and were barely detectable in the rostral and caudal areas of the spinal cord tissue. These results indicated that while most of the untreated DPCs did not remain after transplantation, FGF2 priming slightly but significantly increased the survival rate of DPCs after transplantation.

Next we estimated the minimum number of FGF2-primed DPCs essential for locomotor recovery in SCI rats. Two-way repeated measure ANOVA revealed a significant effect by group (F [3, 17] = 9.623; p < 0.001) and by weeks after injury/transplantation (F [2, 34] = 41.46; p < 0.0001], with interaction between group and the weeks after injury/transplantation (F [6, 34] = 7.474; p < 0.0001]. Post hoc analysis with Tukey’s test indicated that transplantation of DPC-FS at 1 × 10^5^ and 1 × 10^6^ but not at 1 × 10^4^ cells per rat was sufficient to recover the locomotor function of SCI rats (Table 3). Taken together with the limited viability of cells at 7 weeks after transplantation, these results indicate that FGF2 priming would promote substantial numbers of surviving DPCs during the early days after transplantation rather than maintain a small portion of the transplanted cells over a long period, resulting in axonal regeneration in the spinal cord and locomotor function recovery in SCI rats via greater availability of neurotrophic factors.

**Table 3:** Cell-number–dependent effects of DPC-FS transplantation on locomotor recovery (BBB scores).

	Vehicle (n = 5)	1 × 10^4^ (n = 6)	1 × 10^5^ (n = 5)	1 × 10^6^ (n = 5)
1 W	0.1 ± 0.1	0.3 ± 0.1	0.9 ± 0.2	0.7 ± 0.4
3 W	0.9 ± 0.3	0.8 ± 0.3	3.5 ± 1.0*	4.0 ± 0.8**
7 W	0.5 ± 0.3	0.9 ± 0.4	4.4 ± 1.0***	4.9 ± 0.9***

Locomotor function of the hind limbs was evaluated at 1, 3, and 7 weeks after injury and transplantation with the indicated number of DPC-FS. The values are expressed as the mean ± SE. Significant differences were determined using two-way repeated measures ANOVA with a *post hoc* Tukey’s multiple comparison test; *p < 0.05, **p < 0.01, and ***p < 0.001 compared with the SCI/vehicle group.

### Constitutive FGF2 treatment protects DPCs against oxidative-stress–induced death and facilitates locomotor function in the SCI rat after DPC transplantation

**Figure 5:**
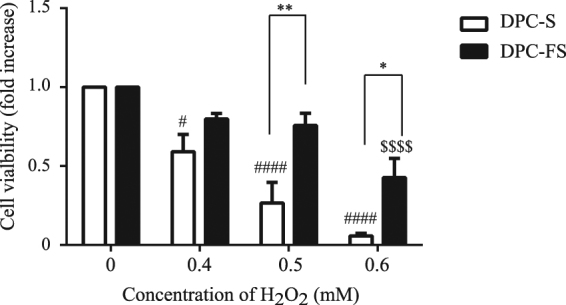
Effect of FGF2 priming on H_2_O_2_-induced DPC death. DPC-S and DPC-FS were exposed to H_2_O_2_ at various concentrations (0, 0.4, 0.5, and 0.6 mM) for 24 hr, followed by MTT assay. The ratios of the absorbance values for the DPCs exposed to H_2_O_2_ were calculated relative to the control (H_2_O_2_-untreated DPCs). The values are expressed as the mean ± SE. Significant differences between the two groups were determined by two-way ANOVA post hoc with Tukey’s multiple comparison test. *p < 0.05, **p < 0.01 vs DPC-S treated with H_2_O_2_ at the same concentration; ^#^p < 0.05, ^###^p < 0.001 vs DPC-S untreated with H_2_O_2_; and ^$$$^p < 0.001 vs DPC-FS untreated with H_2_O_2_, respectively; n = 5.

The increased production of reactive oxygen species (ROS) during SCI has been implicated in neuronal cell death, tissue loss, and neurological dysfunction^19,20^. Therefore, DPCs transplanted into the injury site are exposed to ROS, impairing cell function and decreasing viability. To test whether FGF2 can prevent cell death of DPCs due to oxidative stress, we exposed DPC-S and DPC-FS to hydrogen peroxide (H_2_O_2_) at various concentrations (0, 0.4, 0.5, or 0.6 mM) for 24 hr and assessed cell viability by MTT assay. Two-way ANOVA analysis revealed a significant effect by group (F [1, 8] = 9.182; p < 0.05) and by H_2_O_2_ dose (F [3, 24] = 41.05; p < 0.0001), with interaction between group and H_2_O_2_ dose (F [3, 24] = 3.250; p < 0.05). Post hoc analysis with Tukey’s multiple comparisons test indicated that H_2_O_2_ induced acute apoptosis in cultured DPC-S, in a dose-dependent manner; this observation is consistent with previous reports^21,22^. FGF2 treatment significantly decreased H_2_O_2_–induced DPC death (Fig. 5).

To clarify whether DPCs with ROS-resistant/non-resistant phenotypes would be susceptible to FGF2 withdrawal/supplementation, we examined time-dependent alterations in such variants of DPC-FS and DPC-S without and with FGF2 treatment. A pilot study revealed that the survival of ROS-resistant/non-resistant cells in response to 0.5 mM H_2_O_2_ did not differ from their original after 1–2 days of treatment; however, those phenotypes of DPC-S and DPC-FS were altered from 11 days onwards (Supplementary Fig. S4a). On days 1 and 17 day after cultivation, these DPCs were exposed to H_2_O_2_ at various concentrations (0, 0.4, 0.5, and 0.6 mM) for 24 hr, and cell viability was assessed by MTT assay. Two-way ANOVA analysis revealed a significant effect of treatment duration (DPC-S/FS: F [1, 6] = 5.370, p = 0.0597; DPC-FS/S: F [1, 6] = 14.38, p < 0.01] and H_2_O_2_ dose [DPC-S/FS: F [3, 18] = 53.35, p < 0.0001; DPC-FS/S: F [3, 18] = 29.71, p < 0.0001), with interaction between treatment duration and H_2_O_2_ dose (DPC-S/FS: F [3, 18] = 1.667, p = 0.2095; DPC-FS/S: F [3, 18] = 2.827, p = 0.0678). Post hoc analysis with Tukey’s test indicated that the constitutive but not short-term FGF2 withdrawal and supplementation affected H_2_O_2_ dose-dependent DPC death (Supplementary Fig. S3b).

To test whether ROS-resistant phenotypes are important for DPC transplantation therapy for SCI, first we examined whether the DPCs with therapeutic effects would be susceptible to short-term FGF2 withdrawal/supplementation. DPC-S and DPC-FS were transplanted into the spinal cord injury site of rats after 1 hr with or 1 day without FGF2 treatment (DPC-S/FS^1 hr^ and DPC-FS/S^1 day^, respectively; see details in the Materials and Methods section). Two-way repeated measure ANOVA revealed a significant effect by group (F [3, 27] = 4.966; p < 0.01) and weeks after injury/transplantation (F [2, 54] = 65.66; p < 0.0001], with interaction between group and weeks after injury/transplantation (F [6, 54] = 5.463; p < 0.0005). Post hoc analysis with Tukey’s multiple comparisons test indicated that the therapeutic effects of DPC-S and DPC-FS were not altered by short-term FGF2 supplementation or withdrawal (Table 4).

**Table 4:** Effects of short-term FGF2 withdrawal or supplementation on locomotor recovery in SCI rats after DPC transplantation (BBB scores).

	DPC-S (n = 10)	DPC-S/FS^1 hr^ (n = 5)	DPC-FS (n = 10)	DPC-FS/S^1 day^ (n = 6)
1 W	1.3 ± 0.3	0.2 ± 0.1	0.7 ± 0.3	0.3 ± 0.3
3 W	1.7 ± 0.3	1.6 ± 0.3	3.5 ± 0.6*	3.8 ± 0.7*
7 W	2.6 ± 0.5	2.7 ± 0.6	5.0 ± 0.5***	5.3 ± 0.9***

Locomotor function of the hind limbs was evaluated at 1, 3, and 7 weeks after injury and transplantation of DPCs. The values are expressed as the mean ± SE. For statistical comparison, the data for DPC-S and DPC-FS were replotted from Fig. 2; *p < 0.05; ***p < 0.005 vs SCI/DPC-S (two-way ANOVA, *post hoc* with Tukey’s test).

Next, the effect of DPC-S transplantation on the recovery of function in SCI rats was examined in combination with *i*.*p*. injection of edaravone (ROS scavenger^23^). Two-way repeated measure ANOVA revealed a significant effect by group (F [3, 19] = 22.03; p < 0.0001] and weeks after injury/transplantation (F [2, 38] = 32.19; p < 0.0001], with interaction between group and weeks after injury/transplantation (F [6, 38] = 3.576; p < 0.01]. Post hoc analysis with Tukey’s multiple comparisons test indicated that the locomotor functions of SCI rats (SCI/DPC-S/edaravone) were significantly improved compared to those of SCI/DPC-S and SCI/edaravone rats (Table 5).

**Table 5:** Combined effects of edaravone injection and DPC-S transplantation on locomotor recovery in SCI rats (BBB scores).

	Vehicle (n = 6)	Edaravone (n = 4)	DPC-S (n = 7)	DPC-S + edaravone (n = 6)
1 W	0.3 ± 0.1	0.9 ± 0.6	0.5 ± 0.2	1.4 ± 0.4
3 W	1.2 ± 0.2	1.4 ± 0.2	1.7 ± 0.4	3.6 ± 0.4***
7 W	1.3 ± 0.3	1.8 ± 0.4	2.2 ± 0.4	4.8 ± 0.2***

Locomotor function of the hind limbs was evaluated at 1, 3, and 7 weeks after injury and transplantation of DPC-S, with or without injection of edaravone. The values are expressed as the mean ± SE. ***p < 0.001 vs SCI/vehicle (two-way ANOVA, *post hoc* with Tukey’s test).

Finally, to determine whether the edaravone injection could influence DPC-S survival after cell transplantation, the number of GFP-positive cells was examined in the spinal cords of SCI/DPC-S rats at 7 weeks after injury/transplantation, with or without the *i*.*p*. injection of edaravone. Quantitative analysis revealed that the number of GFP-positive cells increased significantly, by approximately 4.0- to 5.0-fold, with the edaravone injection (with edaravone 100.3 ± 30.1 and without edaravone 21.7 ± 8.1 GFP-positive cells per section, *p* < 0.01; Student’s *t*-test, *n* = 8 and *n* = 5, respectively). Taken together, these results indicate that the ROS-resistant phenotype of DPCs plays an important role in cell therapy.

### Oxidative stress did not influence neurotrophic factor production by dying DPCs

To further assess the initial effects of DPC transplantation, we examined the expression of FGF2 and HGF in the DPCs 1 day after transplantation. Substantial numbers of GFP-positive DPCs produced both FGF2 and HGF at the injury site, regardless of FGF2 priming (Supplementary Fig. S5). To determine whether ROS-induced cell death could influence the production of neurotrophic factors in DPCs, we exposed DPC-S and DPC-FS to H_2_O_2_ and assessed their production of BDNF, GDNF, FGF2, and HGF at various time points. Although some of the cells were dying 24 h after H_2_O_2_ treatment, the production of BDNF in DPC-FS (but not in DPC-S) cells was slightly but significantly increased (Supplementary Fig. S3a and b). Production of the other neurotrophic factors by DPCs was not altered after exposure to H_2_O_2_ (Supplementary Figs S3 and S6).

## Discussion

We found that pre-treatment of DPCs with FGF2 significantly increased locomotor function recovery in SCI rats in response to DPC transplantation. The FGF2 priming protected DPCs from H_2_O_2_-induced cell death *in vitro* and promoted axonal regeneration because of the increased number of DPCs surviving in the spinal cord after transplantation. The transplantation of untreated DPCs significantly increased functional recovery of SCI rats when the transplantation was combined with serial one-week *i*.*p*. injection of the free-radical scavenger edaravone. These results indicate that FGF2 priming improves the survival of DPCs transplanted into the injury site, promoting axonal regeneration and locomotor function recovery in the SCI rat.

Promotion of axonal regeneration is considered as one of the major therapeutic goals of SCI^2^. In the spinal cord of the SCI/DPC-FS, GAP-43–positive regenerative axons were 3.0-fold greater than in the spinal cords of SCI/DPC-S at 1 week after cell transplantation (Fig. 3). These results further suggest that axonal regeneration during the acute/subacute phase of SCI is promoted by transplantation of FGF2-primed DPCs. Pretreatment with FGF2 improved the survival of DPCs after transplantation, judging from the 3.0–4.0-fold increase in the number of DPCs at 7 weeks after transplantation (Fig. 4). In contrast, neither the FGF priming nor the ROS-induced cell death influenced DPC production of neurotrophic factors (Supplementary Figs S2, S3 and S6 and Table 2). Therefore, FGF2 priming increased the number of DPCs surviving, which provided neurotrophic factors to the injury site of the spinal cord after the cell transplantation, resulting in promotion of axonal regeneration (Figs 2 and 3) and recovery of locomotor function (Fig. 2). A recent study suggested that DPCs could also produce inducers (MCP-1/ED-siglec-9) of anti-inflammatory M2-like macrophages. The MCP-1/ED-siglec-9–induced M2-like cells also expressed the neurotrophic factors BDNF, HGF, and VEGF, resulting in the promotion of neurite extension and neuroprotection^24^. Such a mechanism also might be involved in DPC-FS transplantation therapy.

Primary spinal trauma is caused by physical pressures on the spine and generates disruptions in the spinal tissues and blood–spinal cord barriers. Secondary injury follows the primary injury, occurring over a period of several weeks to months^25,26^. Secondary neurodegeneration includes neuroinflammation associated with excitotoxicity and oxidative stress. In this process, inflammatory cells such as microglia and macrophages are activated, infiltrate into the injury site, and generate reactive free radicals and cytotoxic by-products that lead to permanent destruction and loss of spinal cord tissues. Fluid-filled cystic cavities develop and become surrounded by reactive astrocytes and fibroblasts within 2 weeks.

Suppression of secondary injury processes is another therapeutic goal. Minocycline^27^ and molecules secreted from DPSCs^24^ have been reported to alter the active state of microglia/macrophages from proinflammatory and cytotoxic toward anti-proinflammatory and neuroprotective. This shift spares spinal cord gray/white matter-tissues and promotes function recovery in SCI rats. However, a protective effect by DPC transplantation against secondary injury was not clear in the present study. That is, severe cystic cavitation was observed in the spinal cords of all three SCI groups (SCI/DPC-S, SCI/DPC-FS, and SCI/vehicle; Fig. 2). One possible explanation is that a larger number and/or longer-lasting availability of DPCs surviving is required to suppress cystic cavitation than is needed to promote axonal regeneration. Because the effect of DPC transplantation was quite limited for suppressing the activation of microglia/macrophages, irrespective of FGF2 priming (judging from their ameboid-form morphological changes [Fukumitsu *et al*., unpublished observation]). In any case, slight modification of neuroinflammation is not sufficient to improve locomotor function in an SCI model^2^. In addition, *i*.*p*. injection of the radical scavenger facilitated DPC survival in the SCI site but did not affect the recovery of function by the *i*.*p*. injection, itself (Table 5).

ROS are potent apoptosis inducers in various cell types, including DPCs^21,22^. FGF2 prevents ROS-induced death of neurons and cardiomyocytes^28,29^. To our knowledge, little or no information is available concerning the effect of FGF2 on ROS-induced death of DPCs, although FGF2 is proposed to stimulate DPC proliferation and differentiation^16,30^. In the present study, we observed that 1–2 weeks of constitutive FGF2 withdrawal or supplementation alters the resistance of DPC to hydrogen peroxide (Supplementary Fig. S4). Further investigations are necessary to clarify how DPCs become resistant to oxidative stress after FGF2 treatment. Such investigations may include analysis of alterations in gene-expression and/or epigenetic profiles in DPCs upon FGF2 treatment.

Treatment of SCI might be expected to start as soon as possible, since axonal degeneration and tissue destruction of the spinal cord progresses over time after spinal trauma. However, to avoid inflammatory reactions that have cytotoxic effects, the optimal timing of cell transplantation is considered to be the subacute phase of SCI (approximately 7–9 days after SCI in rodents^31,32^). In this study, we demonstrated that FGF2 priming and injection of a radical scavenger affected the survival of DPCs, resulted in improved locomotor function recovery in SCI rats, even if DPCs were transplanted into the injury site immediately after SCI. Contrary to our findings, two previous studies have reported that the DPSC transplantation was effective without FGF2 priming for improving locomotor function recovery of SCI rats^6,33^. A major difference between the previous studies and this study was the cell transplantation method. In the previous studies, DPSCs were also transplanted into intact tissue around the lesion site; thus many DPSCs may have avoided exposure to ROS. Assuming clinical application, we directly injected DPCs into the injury site to avoid the possibility that the needle could create further injury to the parenchyma of the spinal cord.

In most clinical studies, lumbar punctures or intravenous injections are considered safer and thus are the selected methods of cell transplantation. Therefore, it is necessary to examine whether FGF2 priming would be effective for the transplantation of DPCs by these routes. The transplanted DPCs need to migrate toward the lesion site and to survive in the ROS-rich environment; otherwise, they would fail to promote axonal regeneration and locomotor recovery. The FGF2-primed DPCs with ROS-resistant phenotypes will be useful cells for therapy. Although these limitations are considerable, the present study might provide a good standard model for investigating potential molecular targets that protect DPCs exposed to the severe inflammatory reactions following spinal trauma.

## Materials and Methods

All experiments were approved by the Bioethics Committee and the Animal Care and Research Committee of Gifu Pharmaceutical University and Gifu University and were carried out in accordance with the National Institutes of Health guidelines on animal care. All efforts were made to minimize animal suffering and to reduce the number of animals used. In addition, all the methods were performed in accordance with the relevant guidelines and regulations.

### Production of lentivirus expressing GFP

Lentiviruses expressing GFP (lenti-GFP) were produced by calcium phosphate transfection of 293FT cells with pCMV-VSV-G, pCD/NL-BH*DDD, pCD/NL-BH*DDD, and pCS-CPGm plasmid DNAs. At 48 hr after co-transfection, lentivirus-containing supernatant was collected and passed through a 0.45-μm filter and 100-fold concentrated with Amicon^®^Ultra 15 mL (Merck Millipore, Darmstadt, Germany). The titer of filtered and concentrated supernatants (lenti-GFP) was estimated by measuring the number of GFP-expressing cells/total number of HEK293 cells.

### Isolation and culture of human dental pulp cells

Dental pulp was collected from the third molars of patients at Gifu University Hospital, with informed consent from each patient; dental pulp cells (DPCs) were prepared as previously reported^34,35^. Briefly, using a protocol approved by the Institutional Review Board of Gifu University, we collected normal human third molars at the Gifu University Medical Hospital after having obtained informed consent from each patient. The pulp tissues were minced into small clumps and digested with 1 mg/mL collagenase type I (Sigma-Aldrich, Saint Louis, MO, USA) for 30 min at 37 °C. Several colonies were obtained after these small clumps were transferred to culture dishes containing MSCGM medium (Luna, Walkersville, MD, USA). Then, fibroblastic cells (DPCs) growing out of the clumps were expanded in MSCGM (Lonza) medium. The medium was changed every 3 days, and cells were sub-cultured to sub-confluence using TrypLE Express (Thermo Fisher Scientific Inc., Waltham, MA, USA).

After 10 passages, the DPCs (1.5 × 10^5^ cells/3.5 cm dish) were incubated for 2 days with lenti-GFP (2.0 × 10^5^ transduction units). The GFP-labeled cells were selected with puromycin (2.0 μg/mL) for 3 days: During this process, most cells became resistance against the antibiotics, and dying cells were barely detectable. The GFP-labeled DPCs were sub-cultured in 10% fetal bovine serum (FBS) containing alpha MEM medium (10% FBS-αMEM) with or without 10 ng/mL of FGF2 (DPC-S and DPC-FS, respectively). All DPCs were cultured in their respective media and used for the experiments after 5–6 passages. For DPC-FS, FGF2 (10 ng/mL) was supplemented daily to the culture medium.

To evaluate the effect of FGF2 treatment duration, DPC-S and the DPC-FS were independently sub-cultured in 10% FBS-αMEM supplemented daily with and without FGF2 (10 ng/mL) for various times after 6 passages, respectively (DPC-S/FS and DPC-S/FS). The DPCs were passaged every 3 days (at 1.0 × 10^6^ cells/10-cm dish). These DPCs were used for cell transplantation and MTT assay. For cell transplantation especially, DPCs were used after a short period of cultivation in the altered conditions as follows: DPC-S cells were treated with FGF2 (10 ng/mL) for 1 hr after the six passages in their original culture conditions (DPC-S/FS^1 hr^); DPC-FS cells were cultured in 10% FBS-αMEM without FGF2 for 1 day after the six passages in their original culture conditions (DPC-FS/S^1 day^).

### Immunocytochemistry

For immunocytochemistry, DPCs were plated at 2 × 10^4^ cells on coverslips (10 mm diameter, Matsunani Glass, Osaka, Japan) coated with Cellmatrix Type IP (Nitta Gelatin Inc., Osaka, Japan) in 24-well dishes and cultured for 2 days. The DPCs were fixed for 10 min by adding an equal volume of 4% (w/v) paraformaldehyde (PFA) in 0.1 M phosphate buffer (pH 7.4) (4% PFA solution) to the culture medium and post-fixed for 10 min at room temperature (r.t.) with the same fixative solution. After washing with PBS, the cells were permeabilized for 30 minutes at 37 °C with 0.3% (v/v) Triton X-100 in 0.1 M Tris-HCl buffer (pH 7.4). The cells were then treated for 30 min at r.t. with PBS containing 2% (w/v) Block Ace (Dainippon Pharmaceutical, Osaka, Japan) to reduce non-specific antibody binding. They were then reacted with primary antibody at 4 °C overnight. The primary antibodies included rabbit anti-Tuj1 (1:2000, Cell Signaling Technology Inc, Danvers, MA, USA), rabbit anti-GFAP (1:1000, Dako Agilent, Santa Clara, CA, USA), rabbit anti-Sox2 (1:1000, Merck Millipore), rabbit anti-MBP (1:1000, Merck Millipore), mouse anti-GFP (1:1000, Invitrogen, Carlsbad, CA, USA), mouse anti-BDNF (1:1000; Abcam, Cambridge, UK), goat anti-FGF2 (1:1000, R&D Systems Inc., Minneapolis, Minnesota, USA), goat anti-GDNF (1:1000; R&D Systems), and goat anti-HGF antibodies (1:1000, R&D Systems). After washing 3 times for 5 min with PBS, these primary antibodies were visualized with goat anti-mouse, goat anti-rabbit and donkey anti-goat IgG conjugated with Alexa Fluor 488, 546 and 546, respectively (1:1000; Invitrogen) at 4 °C overnight, washed with PBS, and mounted in CC/Mount (Diagnostic BioSystems, Pleasanton, CA, USA). The fluorescent signals were observed with an immunofluorescence microscope (BZ-9000 All-in-One; Keyence, Tokyo, Japan).

### Animal Surgery and DPC Transplantation and i.p. injection of edaravone

Seven-week-old female Wistar rats weighing 120–140 g were obtained from Japan SLC, Inc. (Shizuoka, Japan). The animals were anesthetized using a combination of three anesthetics (medetomidine hydrochloride, 0.375 mg/kg, Kyoritsu Seiyaku, Tokyo, Japan; midazolam, 2.0 mg/kg, Meiji Seika Pharma Co., Ltd, Tokyo, Japan; and butorphanol tartrate, 2.5 mg/kg, Sandoz Ltd., Basel, Switzerland). The spinal cord was completely transected using microsurgical scissors after laminectomy at the level of the 10th thoracic vertebra. After arrest of hemorrhage, the distal stump was carefully lifted up to confirm complete transection, and we ensured that the distal and proximal stumps came into contact when the former stump was returned to its original position. Immediately after surgery, DPCs (1 × 10^6^ cells in 10 μL of PBS per rat, unless otherwise indicated) were transplanted into the gap between the rostral and caudal spinal cord stumps. After cell transplantation and the muscles and skin sutured, the animals were placed in standard cages, allowed free access to food and water, and maintained at 23 ± 2 °C with a 12-hr light/dark cycle. For immunosuppression, the rats were received daily intraperitoneal injections of cyclosporine A (10 mg/kg; LC Laboratories, Woburn, MA, USA). For administration of an radical scavenger, the rats were received twice a daily intraperitoneal injection of edaravone (3 mg/kg; Wako Pure Chemical Industries, Ltd. Osaka, Japan), starting after SCI for 1 week.

### Assessment of Locomotor Function

Locomotor function of both hindlimbs was assessed using the Basso, Beattie, and Bresnahan (BBB) locomotor scale in the open field as described previously^36^. Evaluation was performed 1 day after injury; it was subsequently performed once a week by observers blinded to experimental treatment and was continued for 7 weeks after injury.

### Tissue Preparation for Immunohistochemical Analysis

The animals were deeply anesthetized by an intraperitoneal injection of sodium pentobarbital and then cardio-perfused with 4% PFA solution. The 8 mm-long spinal cord tissues, both 4.0 mm rostral and caudal to the lesion site, were dissected out and postfixed in the same fixative overnight at 4 °C. The tissues were then soaked in cold PBS containing 20% sucrose overnight at 4 °C, and subsequently frozen in embedding compound (Sakura Finetek, Tokyo, Japan). Sagittal serial sections 25-μm thick were prepared with a cryostat (model CM 1850; Leica, Nussloch, Germany), attached to adhesive-coated slides (Matsunami Glass), and dried before being used for immunofluorescence studies.

### Immunohistochemical Analysis

Cryostat sections were fixed with 4% PFA solution for 10 minutes at r.t. and permeabilized for 30 minutes at 37 °C with 0.3% (v/v) Triton X-100 in 0.1 M Tris-HCl buffer (pH 7.4). After washing with PBS, the sections were blocked for 30 min at r.t. in PBS containing 2% Block Ace and then reacted with the diluted primary antibody specific for GFAP (1:1000, Dako Agilent), GAP-43 (1:1000, Merk Millipore), FGF2 (1:1000, R&D Systems) and HGF (1:1000, R&D Systems), overnight at 4 °C. After washing three times with PBS, the sections were incubated with fluorescence-conjugated secondary antibody for 3 hours at r.t. or overnight at 4 °C (Alexa 546-conjugated goat anti-rabbit IgG and donkey anti-goat IgG 1:1000; Invitrogen). The slides were washed with PBS and coverslipped with CC/Mount (Diagnostic BioSystems). Finally, the slides were imaged using an immunofluorescence microscope (BZ-9000 All-in-One; Keyence) and a confocal laser microscope (LSM 710; Carl Zeiss Micro Imaging, Jena, Germany).

### Semi-quantitative analysis on GAP-43-positive fibers and GFP-positive cells

From sagittal serial sections of individual rats at 1 week after SCI, three representative sagittal sections were selected at midline and 200 μm bilateral from midline. After immunostaining using anti-GAP-43 antibodies, the GAP-43–positive areas were examined as follows. An area 0.5-mm–wide was measured 1.0 mm rostral and 1.0 mm caudal to the lesion site, evenly subdivided into 10 bins, and numbered from the ventral to dorsal surface outward. The GAP-43–immunostained area and total area in each bin was independently measured using ImageJ software (NIH, Bethesda, USA).

From sagittal serial sections of individual rats at 7 weeks after SCI, five representative sagittal sections were selected at midline, 200, and 400 μm bilateral from midline. After immunostaining using anti-GFP antibodies, the distribution and number of GFP-positive cells per section were analyzed spinal cord tissues located 2.0 mm rostral and 2.0 mm caudal to the injury site.

### Reverse-transcription (RT)-PCR

Total RNA was isolated from cultured DPCs using Trizol Reagent (Invitrogen). One microgram of total RNA was used for each RT reaction with the PrimeScript RT Reagent Kit (Takara, Ohtsu, Japan) according to the manufacturer’s recommendation. Quantitative PCR (qPCR) was performed with SYBR Premix ExTaq (Takara) and analyzed using the Thermal Cycler Device Real-time System (Takara). Primer sequences used for qPCR are shown in Supplementary Table S1.

### MTT assay

Cell viability was evaluated using the MTT assay as described previously^37^. Briefly, the DPCs were plated (5 × 10^3^ cells/well) in 96-well plates and cultured under the indicated conditions for 2 days; the medium was changed daily. Afterwards, the medium was removed again, and cells were exposed to fresh 10% FBS-αMEM with H_2_O_2_ (0.4, 0.5, or 0.6 mM) for 24 hr. MTT solution (at final concentration of 0.5 mg/mL, Sigma–Aldrich) then was added to the culture, and the cells were incubated for an additional 4 hr. After the crystals were dissolved in HCl/isopropanol, the absorbance at 570 nm was determined using a micro plate reader (model 550, Bio-Rad Laboratories, Hercules, CA, USA). The absorbance data are expressed as relative to that of DPCs cultured under indicated conditions without exposure to H_2_O_2_.

### Statistical Analysis

All numerical data are presented as group mean values with standard error (SE). Statistical analysis of locomotor function was assessed using the BBB scale. Analysis of cell viability data measured by MTT assay was performed by two-way repeated measure ANOVA followed by Tukey’s multiple comparison test. A statistical analysis of Sholl assays was performed using one-way ANOVA followed by Tukey’s multiple comparisons test using GraphPad Prism software (version 6.03, San Diego, CA, USA). Statistically significant differences in the number of GFP positive cells in the fraction of the GAP-43-positive area were determined using Student’s *t*-test.

## Electronic supplementary material

Supplemental Information
