# Supplementary material for: Priming with FGF2 stimulates human dental pulp cells to promote axonal regeneration and locomotor function recovery after spinal cord injury
Source: Sci Rep. 2017 Oct 18;7:13500. doi: 10.1038/s41598-017-13373-5 (PMC5647367; doi:10.1038/s41598-017-13373-5)
Supplement: Supplementary file 1 — Supplemental Information [file 41598_2017_13373_MOESM1_ESM.pdf]

Priming with FGF2 stimulates human dental pulp cells to promote axonal regeneration and locomotor function recovery after spinal cord injury

Kosuke Nagashima[1†], Takahiro Miwa[1†], Hitomi Soumiya[1†], Daisuke Ushiro[1], Tomoko Takeda-Kawaguchi[2], Naritaka Tamaoki[2,3], Saho Ishiguro[1], Yumi Sato[1], Kei Miyamoto[4,5], Takatoshi Ohno[4,6], Masatake Osawa[7], Takahiro Kunisada[8], Toshiyuki Shibata[2], Ken-ichi Tezuka[8], Shoei Furukawa[1], Hidefumi Fukumitsu[1*]

[1]Laboratory of Molecular Biology, Department of Biofunctional Analysis, Gifu Pharmaceutical University, 1-25-4 Daigakunishi, Gifu 501-1196, Japan

[2]Department of Oral and Maxillofacial Science, Gifu University Graduate School of Medicine, 1-1 Yanagido, Gifu 501-1194, Japan

[3]Current address: National Cancer Institute, National Institutes of Health (NIH), Bethesda, Maryland 20892, USA.

[4]Department of Orthopaedic Surgery, Gifu University Graduate School of Medicine, 1-1 Yanagido, Gifu 501-1194, Japan

[5]Current address: Department of Orthopaedic Surgery and Spine Center, Gifu Municipal Hospital, 7-1 Kashima, Gifu 500-8323, Japan

[6]Current address: Department of Orthopaedic Surgery, Gifu Red Cross Hospital, 3-36 Iwakura, Gifu 502-0844, Japan

[7]Department of Regeneration Technology, Gifu University Graduate School of Medicine, 1-1 Yanagido, Gifu 501-1194, Japan

[8]Department of Tissue and Organ Development, Gifu University Graduate School of Medicine, 1-1 Yanagido, Gifu 501-1194, Japan

[*]Correspondence should be addressed to: Dr. Hidefumi Fukumitsu, Laboratory of Molecular Biology, Department of Biofunctional Analysis, Gifu Pharmaceutical University, 1-25-4 Daigakunishi, Gifu 501-1196, Japan; Email: hfukumi@gifu-pu.ac.jp.

Materials and Methods

Preparation of the medium conditioned by DPCs

At the 6th passage, the DPC-S and the DPC-FS were seeded onto 6 cm dishes coated with Cellmatrix Type IP (Nitta Gelatin Inc), at a density of 5×10^5 cells per dish and cultured in the 10% FBS-±MEM supplemented without or with 10 ng/mL of FGF2, respectively. After 2 DIV, the cells were rinsed twice with PBS and the medium was replaced with neuronal culture medium without B27 supplement (Neurobasal medium [Invitrogen] containing 0.5 mM L-glutamine, and 100 U/ml penicillin). After 2 DIV, the medium was collected and filtered through a sterile 0.45 μm pore-size membrane filter (DISMIC-25, Advantec, Tokyo, Japan). The medium was used as conditioned medium for neuron morphological analysis.

Isolation and culture of mouse cortical neurons and morphological analysis

Cortical neurons were prepared from cerebral cortices of embryonic day 15.5 mice and cultured as described previously.[37] In brief, embryos were collected removed from timed-pregnant ddY mice after killed and their cortices were dissected in L-15 medium (Sigma-Aldrich). The cortices were incubated in L-15 medium containing 0.25% trypsin and 0.1% DNase for 15 min at 37°C. After incubation, the digestion was stopped with FBS (HyClone, Thermo Fisher Scientific, Logan, UT, USA), and mechanically dissociated by pipette trituration in DMEM/F-12 (Sigma-Aldrich) containing 10% FBS. The cells were seeded onto round 13 mm coverslips (Matsunami Glass) coated with 0.05 mg/mL poly-L-ornithin (Sigma-Aldrich) in culture 24-wells plates at density of 5×10^4 cells per well. After cell attachment, the medium was replaced by culture medium (Neurobasal medium [Invitrogen] with 2% B27 supplement

[Invitrogen], 0.5 mM L-glutamine, and 100 U/ml penicillin) with lenti-GFP (2.0×10^5 transduction units) under humidified conditions in 95% air and 5% CO_2 at 37°C. After 2 DIV, the medium was replaced with the DPC-cultured medium. The cortical neurons were incubated from 2 to 4 DIV and were processed for immunostaining and morphological analysis.

For morphological analysis, the cultured cortical neurons were immunostained and then processed using a confocal laser microscope (LSM 710; Carl Zeiss) as described previously.[37] Sholl analysis[2] with slight modification was performed for evaluating neuron morphology. In brief, circles with radii of 50 and 100 μm were centered on the cell body, respectively. Then, the number of intersections with both MAP2 and GFP double-positive neurites was recorded. All the measured neurites started at the cell body.

Membrane-based human growth factor antibody array and ELISA

At the sixth passage, the DPC-S and the DPC-FS cells were seeded onto Cellmatrix Type IP-coated 6-cm dishes at a density of 1×10^6 cells per dish and cultured in 10% FBS-±MEM without FGF2. The next day, cells were rinsed twice with PBS and cultured in 2 mL of fresh 10% FBS-±MEM for 2 days. For the microarray, a human growth factor antibody array was purchased from Abcam (ab134002). The cells were harvested with 500 μL of lysis buffer, and cell lysates were prepared for use in the microarray assay, which was performed according to the manufacturer's instructions. Briefly, membranes were incubated with 1× blocking buffer at r.t. for 30 min, followed by overnight individual incubation at 4°C in 1 mL of the lysates from the DPC-S and DPC-FS cells. After washing and incubation with biotin-conjugated anti-cytokine

antibodies at 4°C overnight, the membranes were again washed and then incubated with horseradish peroxidase-streptavidin at 4°C overnight. After washing, the membranes were incubated with the provided detection buffer, and signals were imaged using an LAS3000 mini-CCD camera (Fuji Film, Tokyo Japan).

The amount of BDNF in the cell culture medium and the DPC lysates was measured by a commercial ELISA kit (Promega, Madison, WI, USA) according to the manufacturer's instructions. Briefly, after the cells (1×10^6 cells per 6-cm dish) were cultured in 2 ml of fresh 10% FBS-±MEM for 2 days, cell lysates were harvested with 150 µl of lysis buffer. The cell lysates (diluted 1/4) and the undiluted culture medium were used as samples, which were tested at least in duplicate (n = 4–5).

Exposure to H_2O_2, western blot analysis, and immunocytochemistry

The DPCs were plated at 4×10^5 cells/6-cm dish for western blotting, and at 3×10^4 cells/coverslip coated with Cellmatrix Type IP for immunocytochemistry, in 24-well dishes. They were cultured for 2 days, and the medium was changed daily. The DPCs were then washed twice with PBS and exposed to fresh 10% FBS-±MEM with 0.3–0.5 mM H_2O_2 for 0, 6, and 24 h. A concentration of H_2O_2 was used, which induced approximately 50% DPC-S cell death, and this varied between 0.3 and 0.5 mM depending on the batch of H_2O_2.

Western blotting was performed as previously reported[37]. Briefly, at the indicated time points after exposure to H_2O_2, DPCs were washed with 10 mM Tris-HCl buffer (pH 8.0) containing 150 mM NaCl and then lysed with 200 µl of 20 mM Tris-HCl buffer (pH 7.2) containing 150 mM NaCl, several detergents, and protein inhibitors. The cell lysates were centrifuged, and the protein concentration of each

supernatant was determined with a BCA Protein Assay Kit (Pierce, Rockford, IL). Each sample (for ²-actin, 5 µg; for FGF2, GDNF, and HGF, 10 µg; and for BDNF, 18 µg of protein/lane) was subjected to SDS polyacrylamide gel electrophoresis (PAGE; for BDNF, FGF2, and GDNF, 14%; for HGF and ²-actin, 10%). Proteins were transferred to a polyvinylidene fluoride membrane and blocked with 5% skim milk (Morinaga Milk Products, Tokyo, Japan). Next, the membranes were incubated with primary antibodies (anti-BDNF, 1:1000, Abcam; anti-FGF2, 1:1000, R&D Systems; anti-GDNF, 1:1000, R&D Systems; and anti-HGF antibody, 1:1000, R&D Systems) at 4°C overnight, washed, and then incubated with alkaline phosphatase-conjugated secondary antibody (Promega) at r.t. for 1 hr. Finally, the protein bands were developed with nitroblue tetrazolium and 5-bromo-4-chloro-3-indoryl phosphate p-toluidine salt.

At the indicated time points after exposure to H_2O_2, the DPCs were fixed with 4% PFA solution, and immunocytochemistry was performed as described above.

Supplemental Table S1. Primer sets used in this study

Gene	Forward	Reverse
BDNF	52TCTCGACTGGAGGACCACACTC-32	52CCAACCTGCTCCAGGCTAATCC-32
NT3	52AACGCGATGTAAGGAAGCCAGG-32	52GCTCGGACGTAGGTTTGGGATG-32
GDNF	52TGCAGCTGAGACAACGTACGAC-32	52TCATCAAAGGCGATGGGTCTGC-32
VEGF	52CTCCGAAACCATGAACTTTCTGC-32	52TCGTGATGATTCTGCCCTCCTC-32
GAPDH	52CCGATTTCTCCTCCGGGTGATG-32	52ACCATGTAGTTGAGGTCAATGAAGG-32

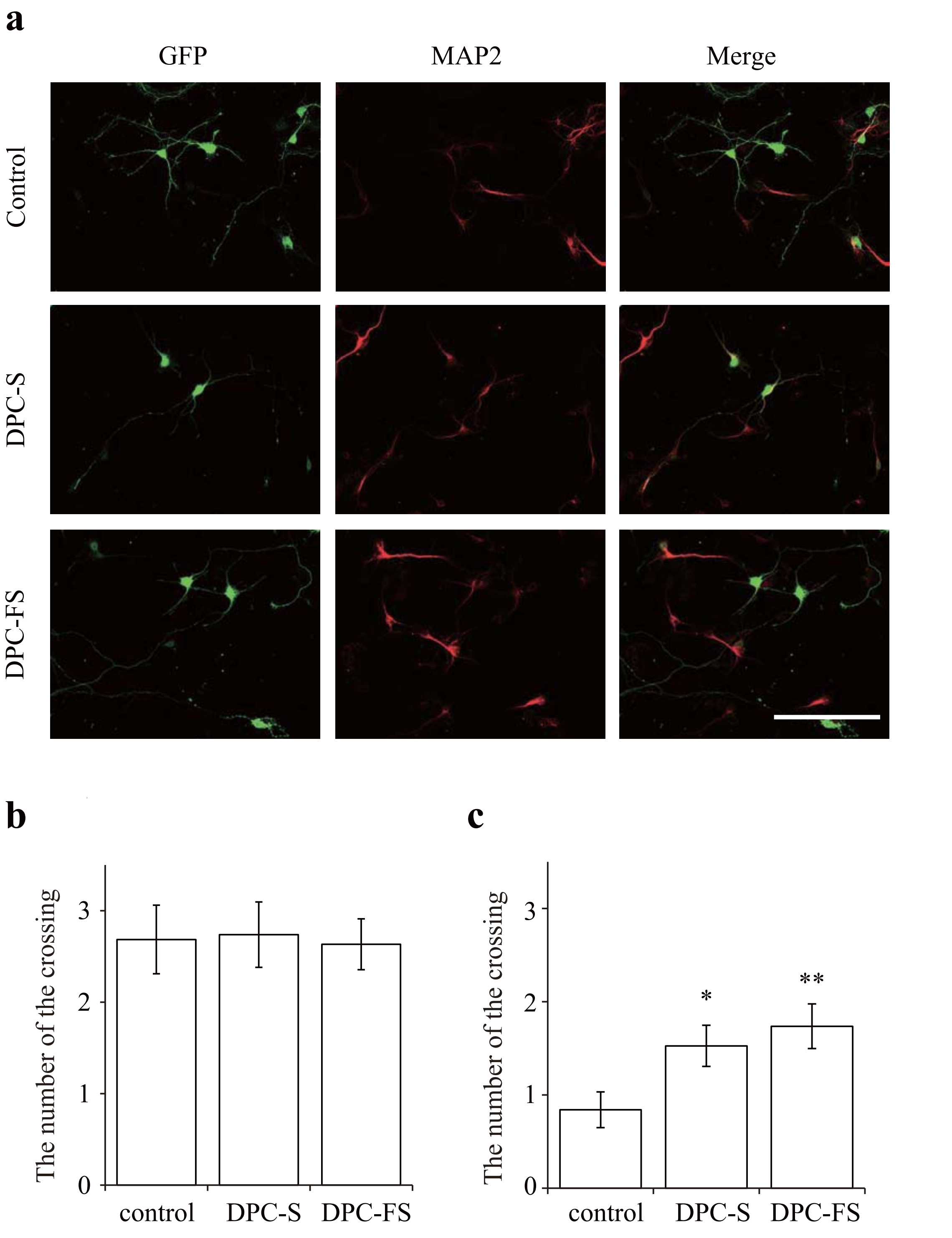

Supplementary Figure S1. Effect of DPC-conditioned medium on the neurite extension in cortical neurons. a. Fluorescence photomicrographs of mouse cortical neurons immunostained with anti-GFP and anti-MAP2 antibodies at 2 days after lentivirus-mediated GFP gene transfer in medium unconditioned or conditioned by DPC-S or DPC-FS. **b, c.** Sholl analysis. Values are expressed as the mean ± SE. Significant differences between the two groups were determined by one-way ANOVA post hoc with Tukey' s multiple comparison test. *$p < 0.05$ and **$p < 0.01$, respectively; n = 19. Note that the effect of conditioned medium was observed at a distance over 100 μm (**c**) but not 50 μm (**b**) from the soma. However, the effect on neurite extension was comparable between the medium conditioned by DPC-S and DPC-FS.

a **b**

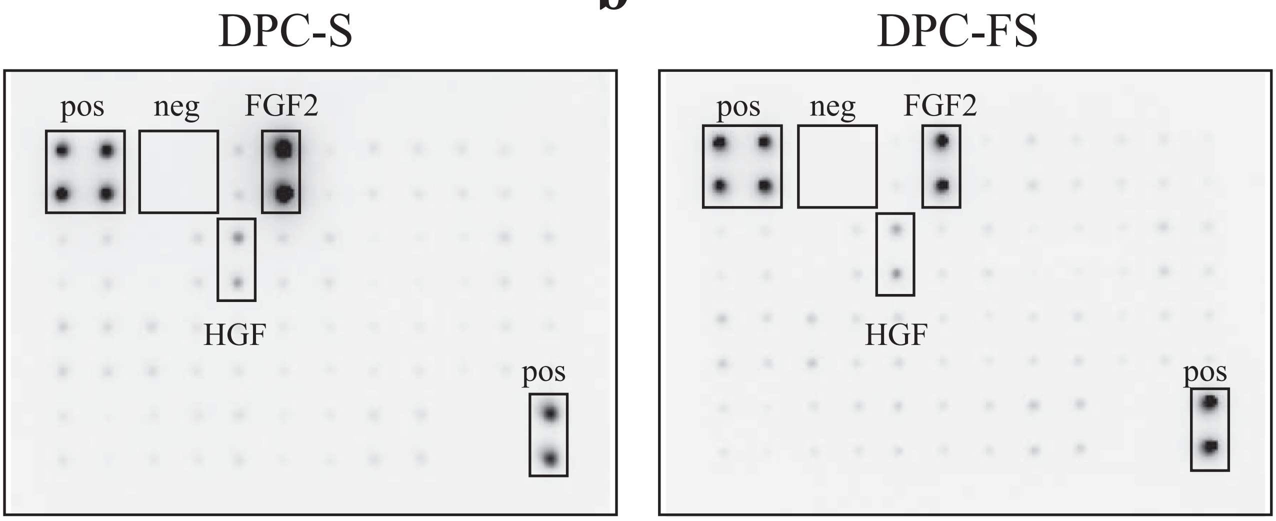

c

	A	B	C	D	E	F	G	H	I	J	K	L
1	pos	pos	neg	neg	AR	FGF2	NGF	EGF	EGFR	FGF4	FGF6	FGF7
2	pos	pos	neg	neg	AR	FGF2	NGF	EGF	EGFR	FGF4	FGF6	FGF7
3	GCSF	GDNF	GM-CSF	HB-EGF	HGF	IGFBP1	IGFBP2	IGFBP3	IGFBP4	IGFBP6	IGF1	IGF1SR
4	GCSF	GDNF	GM-CSF	HB-EGF	HGF	IGFBP1	IGFBP2	IGFBP3	IGFBP4	IGFBP6	IGF1	IGF1SR
5	IGF2	MCSF	MCSFR	NT3	NT4	PDGFRα	PDGFRβ	PDGF-AA	PDGF-AB	PDGF-BB	PIGF	SCF
6	IGF2	MCSF	MCSFR	NT3	NT4	PDGFRα	PDGFRβ	PDGF-AA	PDGF-AB	PDGF-BB	PIGF	SCF
7	SCFR	TGFα	TGFβ	TGFβ2	TGFβ3	VEGF	VEGFR2	VEGFR3	VEGF-D	BLANK	BLANK	pos
8	SCFR	TGFα	TGFβ	TGFβ2	TGFβ3	VEGF	VEGFR2	VEGFR3	VEGF-D	BLANK	BLANK	pos

Supplementary Figure S2. Growth factor antibody array. Lysates from DPC-S (**a**) and DPC-FS (**b**) cells were incubated with a membrane containing 41 antibodies, with each antibody recognizing an individual growth factor. **c.** The localization of the antibodies spotted on a human growth factor antibody array.

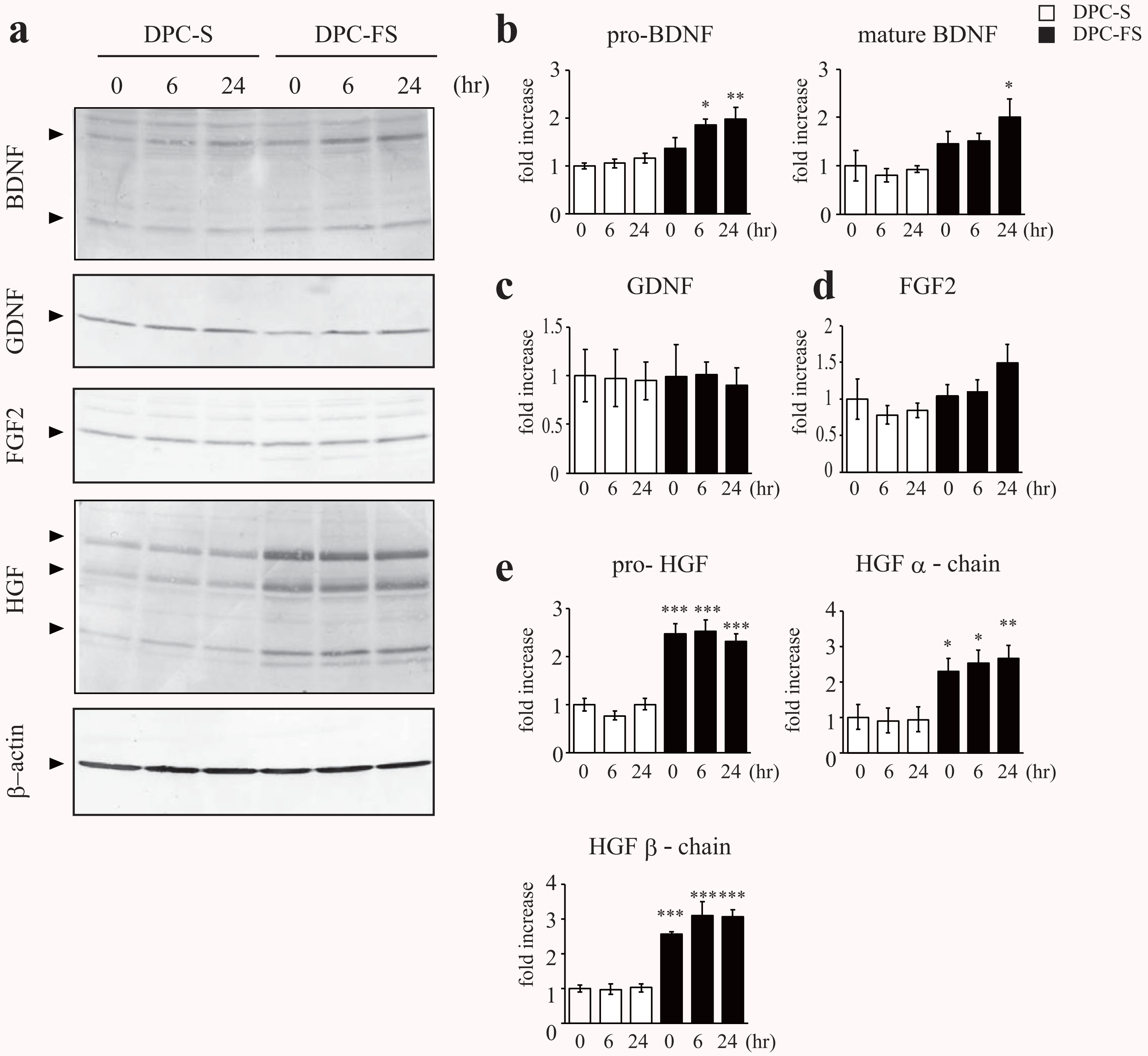

Supplementary Figure S3. Production of neurotrophic factors by the DPCs after exposure to 0.3 mM H_2O_2. a. Cell lysates from DPC-S and DPC-FS were subjected to PAGE and transferred onto membranes for western blotting with anti-BDNF, anti-FGF2, anti-GDNF, anti-HGF, and anti-β-actin antibodies. **b–e.** The intensity of the target bands (indicated by arrowheads in **a**) for each neurotrophic factor was densitometrically quantified, and the ratio of the intensity of each target protein/β-actin was expressed as a fold increase of the average value of individual target bands after exposure to H_2O_2 compared with at 0 hr. The neurotrophic factor target bands were as follows: **b.** BDNF and pro BDNF, at 14 and 30 kDa, respectively; **c.** GDNF, at 18 kDa; **d**. FGF2, at 18 kDa; **e.** pro-HGF and α- and β-chains of HGF, at 95, 60, and 34 kDa, respectively. The values are expressed as the mean ± SE (n = 5). Significant differences from the value of individual target bands at 0 h after exposure to H_2O_2 were determined by one-way ANOVA followed by Tukey' s test; *p < 0.05, **p < 0.01, and ***p < 0.001, respectively.

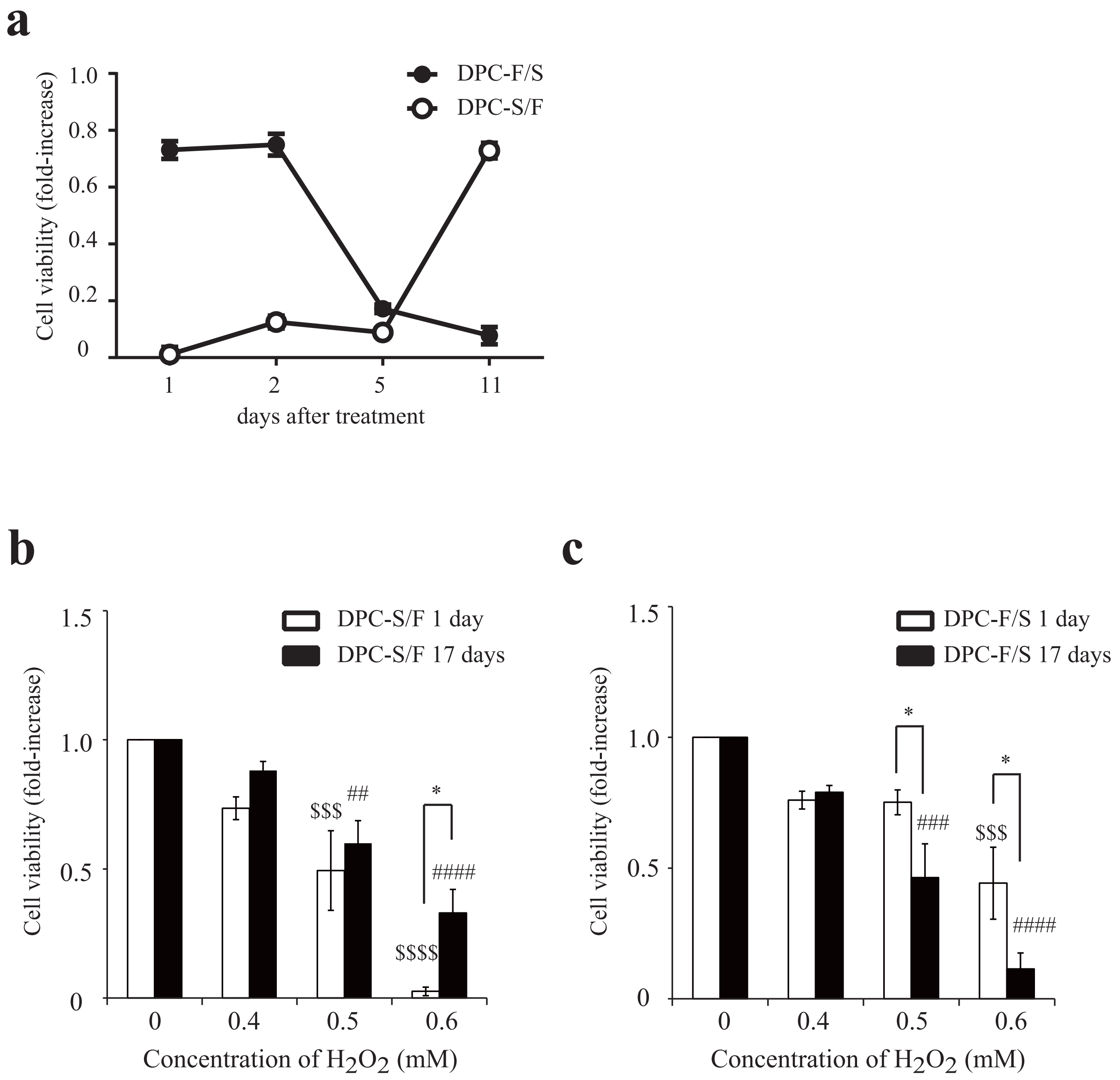

Supplementary Figure S4. Time-course effect of FGF2 supplement and withdrawal on the H_2O_2-induced DPC death. DPC-S and DPC-FS were independently cultured in medium supplement with and without FGF2, respectively (DPC-S/FS, and DPC-S/FS). **a.** At the indicated days after cultivation, the DPCs were exposed to 0.5 mM H_2O_2 for 24 hrs, followed by MTT assay. **b.** On days 1 and 17 after cultivation, the DPCs were exposed to H_2O_2 at various concentrations (0, 0.4, 0.5, and 0.6 mM) for 24 hrs, followed by MTT assay. The ratios of the absorbance values for the DPCs exposed to H_2O_2 were calculated relative to controls (H_2O_2-untreated DPCs). The values are expressed as the mean ± SE. Significant differences between the two groups were determined by two-way ANOVA post hoc with Tukey' s multiple comparison test. *p < 0.05 vs DPC-S treated with H_2O_2 at the same concentration; $^{\$\$\$}p < 0.005$, $^{\$\$\$\$}p < 0.001$ vs DPC-S/FS day 1 or DPC-FS/S day 1 treated with 0 mM H_2O_2; and $^{\#\#}p < 0.01$, $^{\#\#\#\#}p < 0.001$ vs DPC-S/FS day 17 or DPC-FS/S day 17 treated with 0 mM H_2O_2, respectively; n = 4.

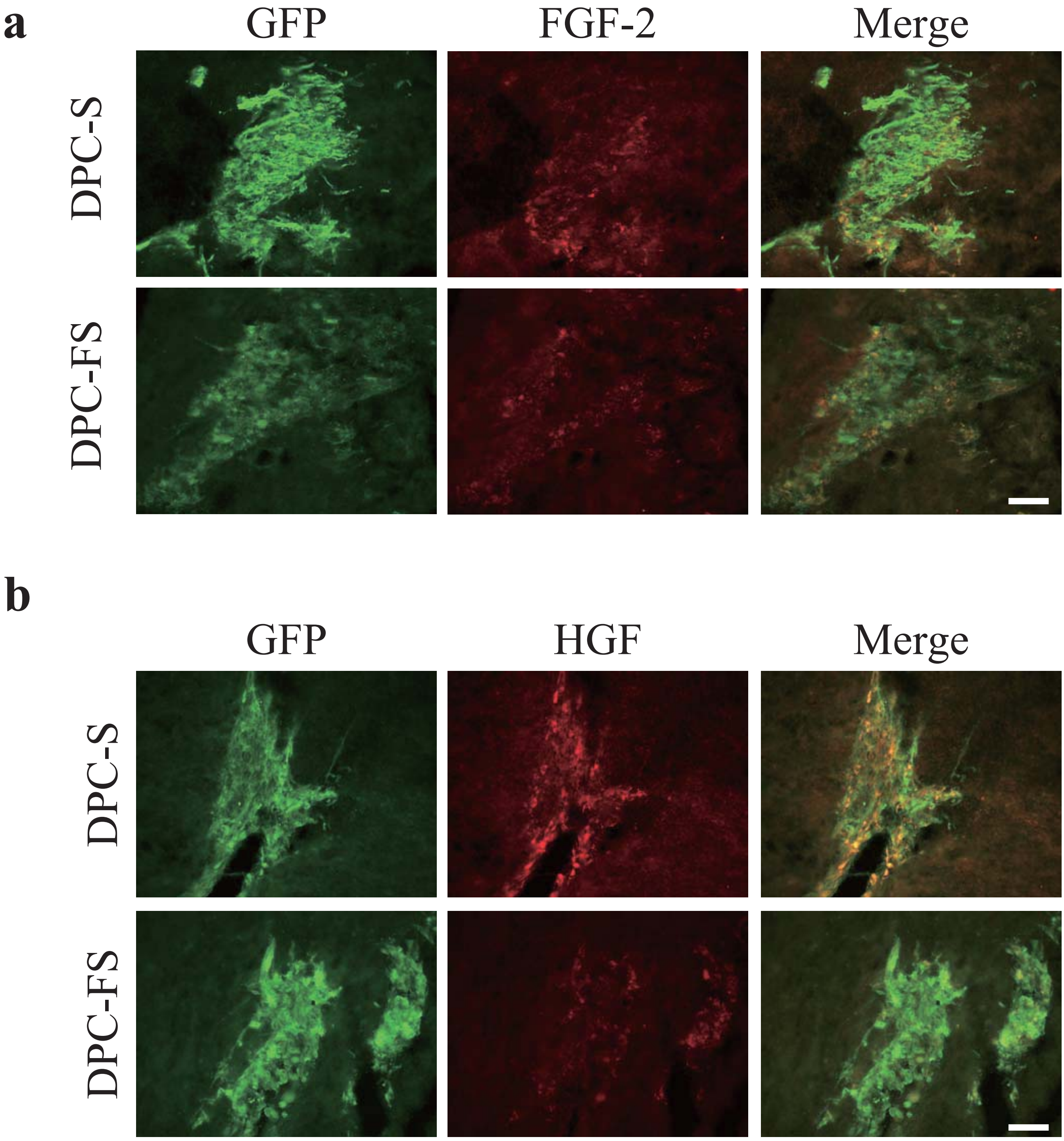

Supplementary Figure S5. Production of neurotrophic factors by DPCs in the spinal cord injury site at 24 hr after transplantation. Fluorescence photomicrographs of sagittal spinal cord sections with SCI/DPC-S or SCI/DPC-FS transplantation. Sections immunostained for GFP (green) and neurotrophic factors (red) were merged. **a.** FGF2 and **b.** HGF. Note that substantial numbers of GFP-positive DPC-S and DPC-FS cells produced FGF2 and HGF. Scale bar, 100 μm.

a

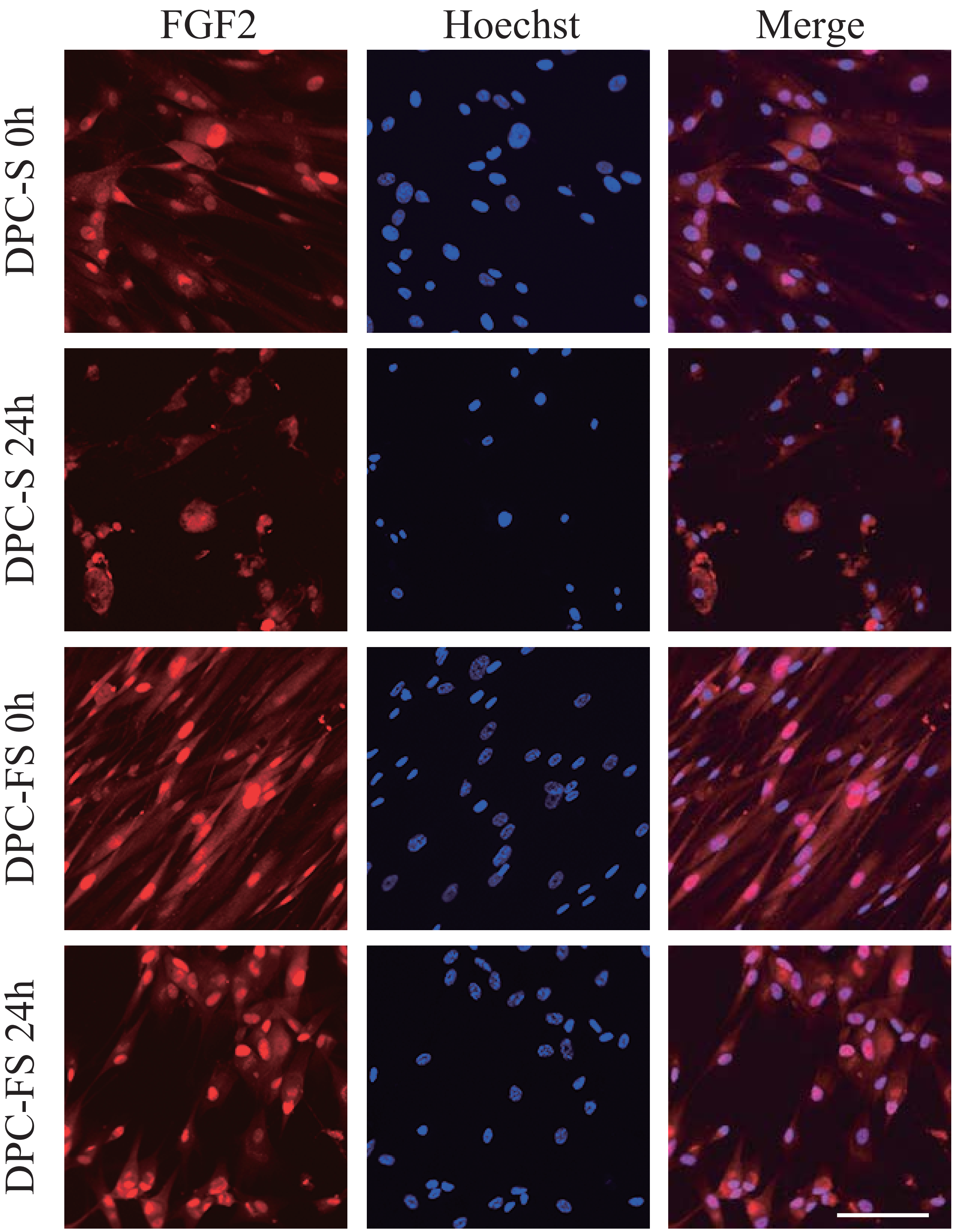

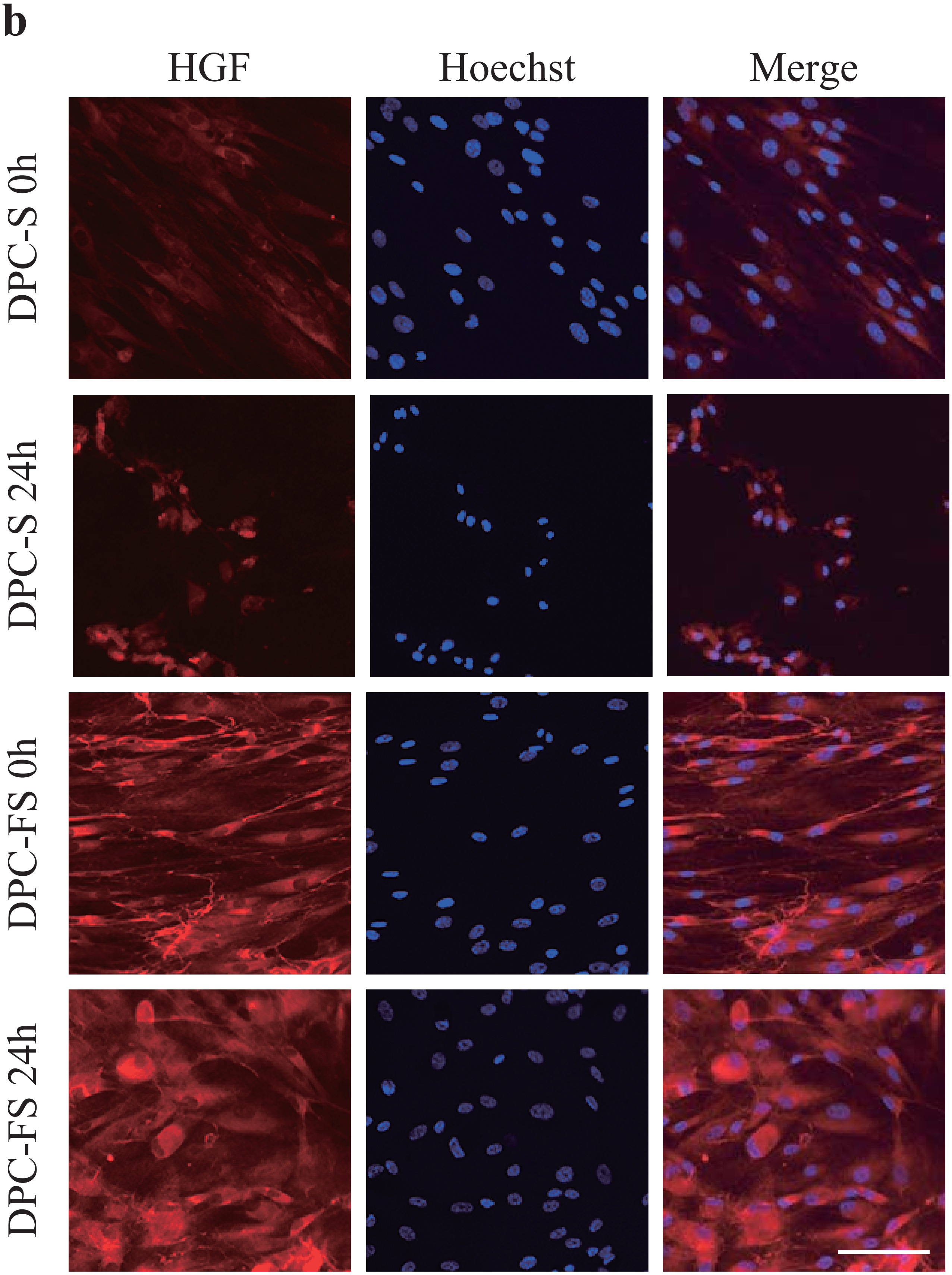

Supplementary Figure S6. Production of neurotrophic factors by cultured DPCs at 0 and 24 h after exposure to 0.3 mM H_2O_2. Cultured DPCs were characterized immunohistochemically using antibodies against FGF2 (red; a) or HGF (red; b). Nuclei were stained with Hoechst 33342 (blue). Note that the cell density of DPC-S was reduced at 24 h after exposure to H_2O_2; however, the production of FGF2 and HGF was not altered after H_2O_2 treatment. Scale bar, 100 µm.
